# Single-Anchor UWB Localization Using a Switched Directional Antenna Array and First-Path Power

**DOI:** 10.3390/s26217002

**Published:** 2026-11-05

**Authors:** Yunsu Bae, Byung-Jun Jang

**Affiliations:** Department of Electrical Engineering, Kookmin University, Seoul 02707, Republic of Korea; bys705@kookmin.ac.kr

**Keywords:** direction estimation, first-path power, single-anchor localization, switched-beam antenna, UWB

## Abstract

IEEE 802.15.4z High-Rate Pulse (HRP) Ultra-Wideband (UWB) wireless technology supports centimeter-level ranging and is widely utilized for indoor positioning. Conventional single-anchor systems typically combine two-way ranging (TWR) for distance measurement with phase difference of arrival (PDoA) processing for direction estimation. However, PDoA accuracy depends strongly on the array aperture, angle of arrival, and received signal quality, which inherently limits the effective positioning range of the system. To address this limitation, this paper proposes a single-anchor positioning system based on an N-element circular array of directional antennas, in which a single UWB transceiver sequentially switches among the antenna elements. The anchor determines the distance via double-sided two-way ranging (DS-TWR) and estimates the direction based on the relative power between channels, obtained by measuring the first-path power of each antenna channel. Experimental evaluations conducted in a 12.8 m × 22.8 m indoor hall resulted in median and 90th-percentile direction errors of 1.93° and 2.66°, respectively, and median and 90th-percentile positioning errors of 0.24 m and 0.36 m, respectively.

## 1. Introduction

Interest in positioning and location-based services using ultra-wideband (UWB) technology has grown substantially [1]. In 2019, Apple introduced UWB technology in the iPhone 11 to enable precise spatial interaction with nearby UWB devices [2]. UWB has subsequently been adopted in applications such as smartphone-based automotive digital keys and peer-to-peer payment services [3,4]. It is now being utilized in a wide range of applications, including automotive systems, retail, smart factories, and smart homes.

UWB is a wireless communication technology that supports both data transmission and high-accuracy distance measurement. The high-rate pulse (HRP) UWB physical layer specified in IEEE 802.15.4z uses a bandwidth of 499.2 MHz and transmits pulses with a duration of approximately 2 ns [5]. Owing to this high temporal resolution, the distance between UWB devices can be estimated with centimeter-level accuracy based on time of flight (ToF) [6]. However, two-way ranging (TWR) provides only the relative distance between two devices. Therefore, conventional two-dimensional range-only localization typically requires at least three UWB anchors installed at known fixed positions. Each anchor measures its distance to the target tag, and the tag position is then calculated through trilateration using the known anchor locations [7].

**Figure 1:**
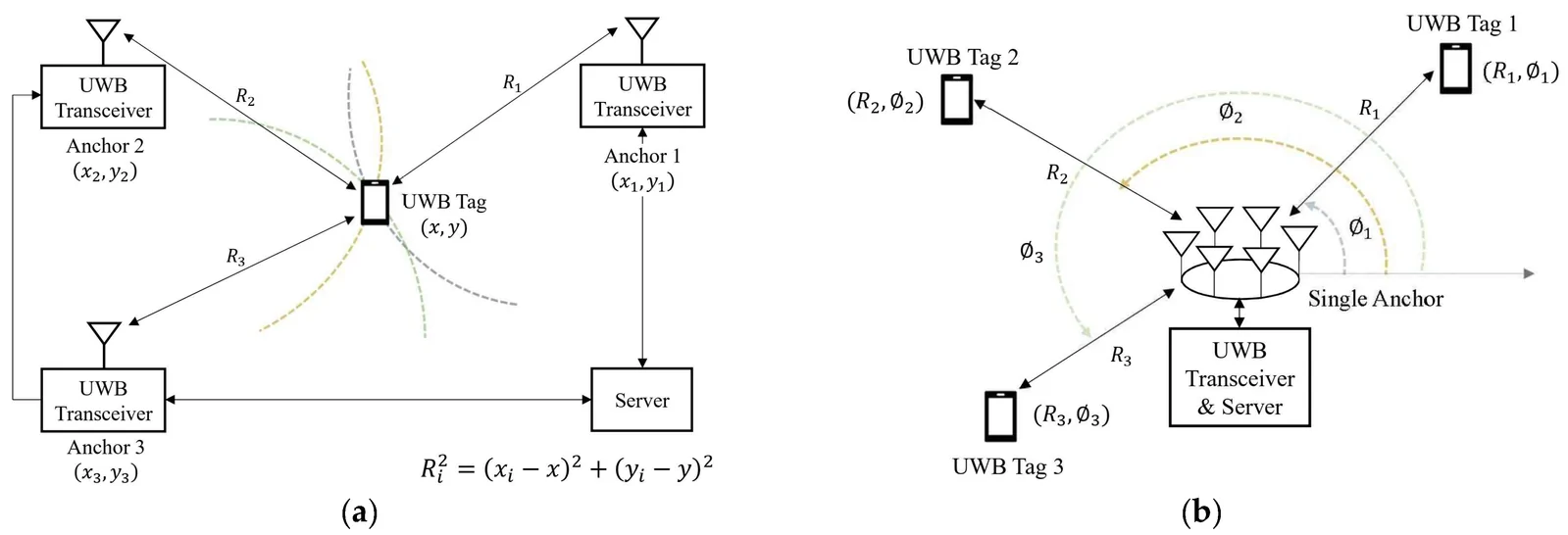
UWB RTLS configurations: (**a**) conventional multi-anchor system and (**b**) proposed single-anchor system.

Based on this principle, conventional UWB real-time location system (RTLS) architectures, as illustrated in Figure 1a, have been extensively studied [8,9]. However, such systems require multiple anchors to be installed at predefined locations and connected to a processing unit through wired or wireless links. To reduce this infrastructure requirement, various single-anchor localization approaches have been investigated. A major class combines UWB ranging with phase-based direction estimation using phase difference of arrival (PDoA) or angle of arrival (AoA) measurements [10,11,12,13,14,15,16]. These methods can provide accurate directional information; however, their performance depends on the array geometry and the consistency of inter-channel phase measurements, and phase calibration, synchronization, or ambiguity-resolution processing may be required. In addition, linear-array configurations exhibit reduced angular sensitivity near the array axis, often requiring additional baselines or more complex array geometries for broader directional coverage.

Alternative approaches exploit UWB CIR characteristics [17,18,19,20,21]. Others combine UWB measurements with auxiliary visual, inertial, odometric, or motion information [22,23,24,25,26,27]. These methods can reduce the required localization infrastructure but may require environment-dependent models, statistical processing, or additional sensors. Directional antenna arrays have also been investigated for mono-station and single-anchor localization. Sun et al. [28] combined ToF ranging with monopulse angle estimation using overlapping antenna beams. Direction measurements were acquired in a dedicated interval by switching among antennas and measuring pulse amplitudes, followed by antenna-pair lookup-table processing. Qing et al. [29] developed a six-element sectored UWB array with overlapping patterns for 360° coverage and described direction estimation by comparing received pulse amplitudes with known antenna gain responses. For switched circular arrays, Kawdungta et al. [30] compared four-, five-, six-, and eight-element configurations at 2.4–2.5 GHz, with MUSIC-based direction estimation and Friis-based range estimation from received signal strength. Lian et al. [31] used a motor-driven platform carrying two UWB modules to obtain full-coverage three-dimensional localization.

This paper proposes a single-anchor UWB localization system consisting of a single UWB transceiver, an RF switch, and six directional antennas uniformly arranged around the anchor. The transceiver sequentially selects the antenna channels through the RF switch, and one DS-TWR exchange is performed for each active antenna. In each DS-TWR exchange, the packet timestamps are used to estimate the tag range, while the first-path power extracted from the CIR of the Final packet exchanged during the same DS-TWR round provides the received-power information for direction estimation. After all antenna channels have been measured, the range measurements obtained from all antenna channels are averaged to obtain the final range, and the first-path powers are used to weight the antenna pointing vectors to estimate the tag direction. Both range and direction measurements are therefore obtained within the same DS-TWR exchanges, without a separate direction-measurement sequence. The vector calculation uses the known antenna pointing directions. Using first-path power for the direction weights reduces the contribution of temporally separated multipath components compared with using total CIR power. Using received power instead of inter-channel phase differences for direction estimation, the proposed system simplifies the phase synchronization and calibration required among antenna channels. In addition, because all antennas share a single UWB transceiver, multiple receivers are not required, reducing the power consumption and system size. Furthermore, estimating the two-dimensional tag position using a single anchor simplifies the system configuration and installation compared with conventional multi-anchor systems. The conceptual architecture of the proposed single-anchor system is illustrated in Figure 1b.

The remainder of this paper is organized as follows. Section 2 compares the theoretical performance of typical PDoA array configurations and the proposed power-based direction estimation method through Cramer–Rao lower bound (CRLB) analysis. Section 3 describes the underlying concept and architecture of the proposed system. Section 4 presents the system implementation and scheduling. Section 5 describes the experimental setup and analyzes the localization results, and Section 6 concludes this paper.

## 2. Comparison of Link Characteristics

UWB-based single-anchor localization systems typically measure distance through TWR and estimate the tag’s direction from phase differences between signals received by the antenna array. In this section, we derive the CRLBs for representative PDoA array configurations and the proposed power-based direction estimation method and compare the resulting two-dimensional position error bounds for different array configurations.

The CRLB for PDoA-based angle estimation is given by [32]

(1) $${CRLB}_{PDoA}\left(\theta \right)=\frac{6}{N_{eff}\rho \left(r\right)k^{2}d_{a}^{2}N\left(N^{2}-1\right)\cos^{2}\theta }\;,$$

where $N_{eff}$ is the effective number of independent observations used for phase estimation, ρ(r) is the received SNR at distance r, *k* = 2π/*λ* is the wavenumber, with λ denoting the signal wavelength, $d_{a}$ is the antenna spacing, N is the number of antenna elements, and θ is the angle of incidence measured from broadside. According to (1), the Fisher information increases with the number of antenna elements and the array length. However, as the incident signal approaches a direction parallel to the array axis, $\cos^{2}\theta$ approaches zero, increasing the angle estimation error. Therefore, increasing the number of elements in a linear array does not eliminate the performance degradation along the array axis.

In contrast, the proposed UWB power-based direction estimation method uses differences in received power among directional antennas arranged in a circle. The received power measured at the i-th antenna, for i=0, …, N−1, can be expressed as

(2) $${\hat{P}}_{i}\left(\theta ,r\right)=A\left(r\right)G_{i}\left(\theta \right)+n_{i},$$

where A(r) is the received power accounting for path loss at distance r and receiver gain, $G_{i}(\theta )$ is the gain of the i-th antenna, and $n_{i}$ is the power measurement error with variance $\sigma_{P,i}^{2}$. For the CRLB analysis of this power-based direction estimation method, the azimuth radiation pattern of each antenna is modeled using the following ideal Gaussian function:

(3) $$G_{i}\left(\theta \right)=G_{max}\exp\left[-4\ln2{\left(\frac{{∆}_{i}\left(\theta \right)}{\Theta_{HPBW}}\right)}^{2}\right],$$

(4) $${∆}_{i}\left(\theta \right)=atan2\left[\sin\left(\theta -\varphi_{i}\right),\cos\left(\theta -\varphi_{i}\right)\right],$$

and

(5) $$\varphi_{i}=\frac{2\pi i}{N},$$

where $G_{max}$ is the maximum antenna gain, ${∆}_{i}\left(\theta \right)$ is the angular difference between the tag direction and the antenna pointing direction wrapped to the interval [−π, π], $\Theta_{HPBW}$ is the half-power beamwidth, and $\varphi_{i}$ is the pointing direction of the i-th antenna. The CRLB for power-based angle estimation is then given by

(6) $${CRLB}_{Power}\left(\theta \right)={\left[\sum_{i=0}^{N-1}\frac{1}{\sigma_{P,i}^{2}}{\left(\frac{\partial P_{i}\left(\theta ,r\right)}{\partial \theta }\right)}^{2}\right]}^{-1}$$

and

(7) $$\sigma_{P,i}^{2}=\frac{1+2\rho_{i}(\theta ,r)}{N_{eff}\rho_{i}^{2}(\theta ,r)},$$

where $P_{i}\left(\theta ,r\right)=A\left(r\right)G_{i}\left(\theta \right)$ is the received power at the i-th antenna, and $\rho_{i}(\theta ,r)$ denotes the corresponding received SNR. As shown in (6) and (7), the performance of power-based direction estimation is determined by the square of the rate of change of the received power with respect to angle at each antenna and by the variance of the received-power measurement error. With appropriately designed antenna radiation patterns and angular spacing, neighboring antennas provide directional information even when the slope of an antenna’s radiation pattern is small near its boresight, allowing the CRLB to remain constant over the full azimuth range. Antennas facing away from the tag receive little signal and thus have a low SNR, which increases $\sigma_{P,i}^{2}$ and reduces their contribution to the overall CRLB.

To compare the effect of direction estimation errors on the final two-dimensional position estimate for each method, the angular CRLB can be converted into a position error bound. The two-dimensional position error bound is given by

(8) $$PEB\left(r,\theta \right)=\sqrt{\sigma_{r}^{2}+r^{2}CRLB_{\theta }\left(r,\theta \right)},$$

where $\sigma_{r}$ is the standard deviation of the TWR ranging error, and $r^{2}CRLB_{\theta }$ is the lower bound on the variance of the position error due to angle estimation errors. Using this position error bound, we analyzed the CRLB-based position error bounds for four configurations: (1) PDoA with a two-element uniform linear array (ULA), (2) PDoA with a six-element ULA, (3) PDoA with a six-element planar array comprising three two-element baselines, and (4) the proposed power-based direction estimation method with a six-element antenna array. The PDoA antennas were assumed to be omnidirectional with a gain of 0 dBi and an element spacing of λ/2. The three baselines of the planar PDoA array were oriented 120° apart. The proposed power-based direction estimation system was modeled using six Gaussian beams, each with a half-power beamwidth of 60°, corresponding to a maximum gain of 7.51 dBi, and arranged at 60° intervals.

**Figure 2:**
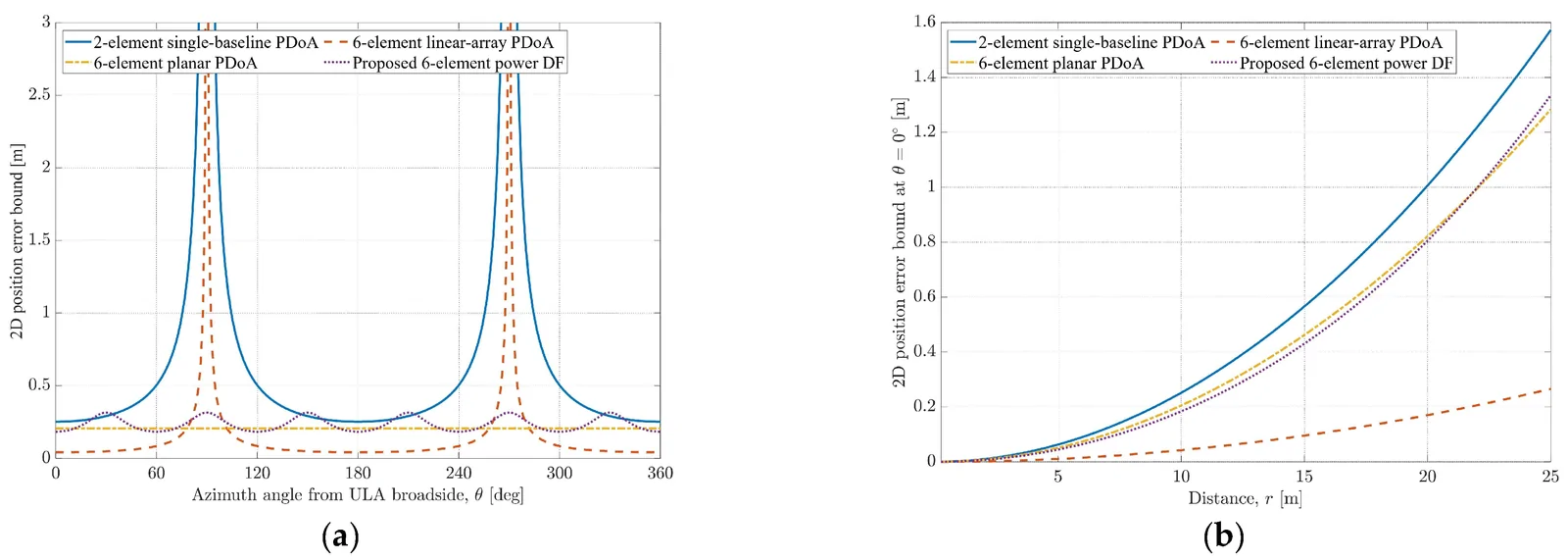
CRLB-based 2D position error bounds versus (**a**) azimuth at r=10 m and (**b**) distance at θ=0°.

Figure 2 compares the position error bounds, and the common parameters used in the analysis are listed in Table 1. Figure 2a shows the position error bounds as a function of angle at a fixed distance of r=10 m. For PDoA with two-element and six-element ULAs, the position error bounds increase sharply near 90° and 270°. This is because signals arriving from these directions are parallel to the array axis, reducing the sensitivity of the phase difference to changes in angle. The six-element linear array provides a lower error bound in other directions owing to its larger number of antennas and greater array length, but the performance degradation along the array axis remains. Assuming that the information from all baselines is correctly combined without phase ambiguity and that no additional phase errors are present, the six-element planar PDoA configuration provides a constant error bound across azimuth angles by combining information from three differently oriented baselines. The proposed power-based direction estimation method exhibits periodic variations in the error bound due to the overlap of the Gaussian radiation patterns but maintains a constant position error bound over the full azimuth range. Figure 2b shows the position error bounds as a function of distance under broadside incidence. The six-element linear PDoA configuration provides the lowest error bound owing to its large array aperture. The proposed power-based method exhibits similar performance to the six-element planar PDoA configuration over the considered distance range, while PDoA with a two-element ULA has the highest error bound. The results in Figure 2 show that the proposed power-based direction estimation method maintains a constant position error bound over the full azimuth range without using inter-channel phase differences. Its error bound is also comparable to that of the ideal three-baseline planar PDoA configuration over the distance range considered.

**Figure 3:**
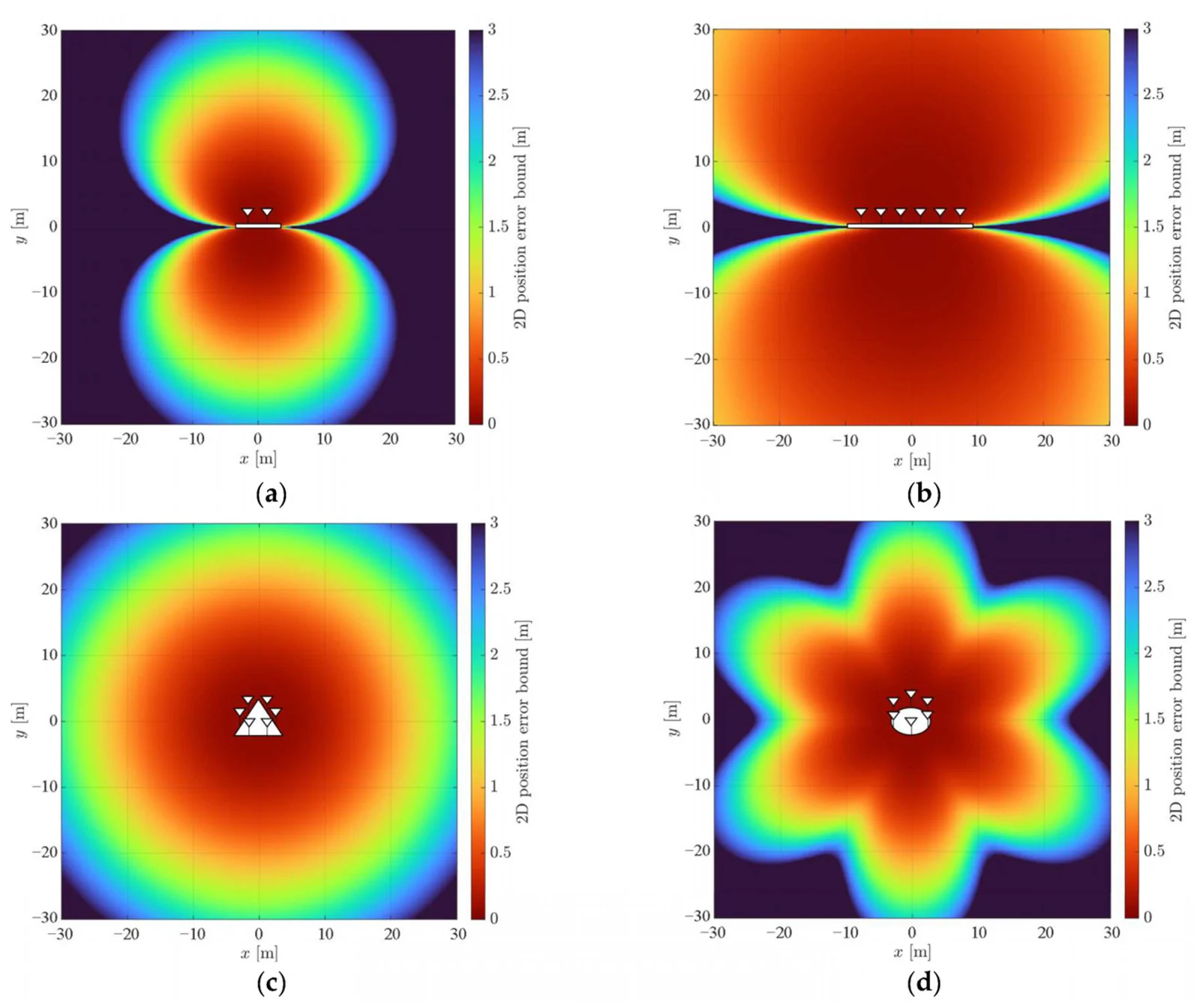
CRLB-based 2D position error maps for (**a**) two-element linear PDoA, (**b**) six-element linear PDoA, (**c**) six-element planar PDoA, and (**d**) the proposed six-element power-based array.

Next, as shown in Figure 3, two-dimensional position error bound maps were calculated under the same link and array conditions to examine the spatial distribution of position error bounds for each array as both distance and azimuth angle vary. To compare only the direction estimation methods, the ranging error was assumed to be zero. The common parameters used to generate the maps for all configurations are listed in Table 1. The two-element ULA-based PDoA configuration in Figure 3a provides a low position error bound in the broadside direction, but the bound increases substantially for signals arriving parallel to the array axis. The six-element ULA-based PDoA configuration in Figure 3b provides lower error bounds in most directions than the configuration in Figure 3a because of its larger number of antenna elements and greater array aperture. However, since it still uses a single baseline, the performance degradation along the array axis remains. The six-element planar PDoA configuration in Figure 3c combines the Fisher information from three baselines oriented 120° apart, resulting in an error bound that does not vary with azimuth under ideal conditions. The proposed power-based direction estimation method in Figure 3d uses six directional antennas arranged at 60° intervals and maintains a constant position error bound over the full azimuth range. These results represent theoretical error bounds under line-of-sight (LoS) conditions with additive white Gaussian noise (AWGN). In a practical PDoA system, residual phase offsets in the receive paths, inter-channel phase calibration errors, phase synchronization errors between receivers, and ambiguity between symmetric angle candidates must be considered and may lead to larger errors. To quantify the effect of residual phase offsets in this comparison, static residual channel offsets were modeled as independent zero-mean variables with equal variance. The corresponding residual phase-difference standard deviation, $\sigma_{∆\varphi }$, was set to 2°, 5°, and 8° for sensitivity analysis. The thermal-noise model and link parameters were unchanged. First-order error propagation through the ideal PDoA phase model gives the local mean-square angular error

(9) $${MSE}_{\theta ,PDoA}\left(r,\theta \right)\approx {CRLB}_{PDoA}\left(\theta \right)\times \left[1+N_{eff}\cdot \rho \left(r\right)\cdot \sigma_{∆\varphi }^{2}\right].$$

**Figure 4:**
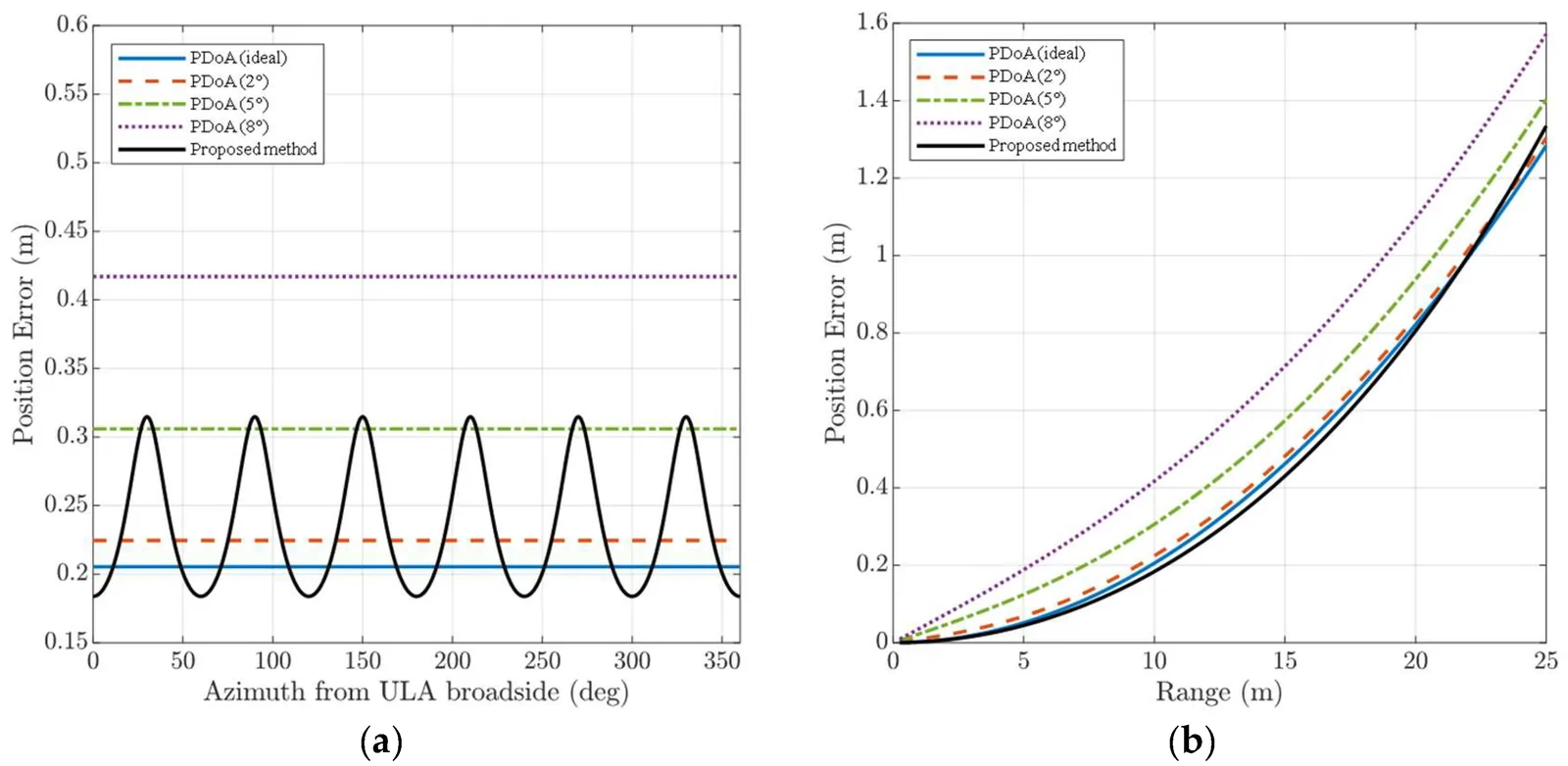
Comparison of theoretical positioning errors for the proposed method and planar PDoA with and without residual phase offsets: (**a**) positioning errors versus azimuth at a distance of 10 m and (**b**) positioning errors versus distance at an azimuth of 0°. The proposed method is evaluated using its CRLB-based bound, while the planar PDoA results are obtained from first-order RMS error predictions.

Here, ${CRLB}_{PDoA}$ denotes the corresponding ideal angular bound of the considered PDoA configuration. The additional term represents the mean squared angular bias over the residual-offset ensemble. Substituting this angular MSE for the angular variance in (8) gives the local RMS position-error prediction. Figure 4a shows that residual phase offsets increase the planar PDoA error level while its azimuthal variation remains negligible under the assumed array model. This follows from the three equally weighted baselines, whose combined angular sensitivity does not depend on azimuth. The power-based bound varies periodically as the overlap of the antenna patterns changes with direction. For a residual phase-difference standard deviation of 2°, planar PDoA gives a lower prediction near the maxima of the power-based bound, whereas the 8° PDoA prediction remains higher over the full azimuth range. Figure 4b shows increasing position error with distance for both methods. At 10 m and 0° azimuth, the planar PDoA prediction increases from 0.205 m without residual offsets to 0.225 m, 0.306 m, and 0.417 m for residual phase-difference standard deviations of 2°, 5°, and 8°, respectively. Residual offsets introduce an angular-error contribution that does not decrease with the effective observation count. Its contribution to position error grows with range, while the thermal-noise contribution also increases as the received SNR decreases. The relative increase over the ideal PDoA prediction is therefore larger at shorter ranges in the considered model.

In particular, PDoA with multiple antennas requires multiple receivers to measure the phases of the signals received at each antenna simultaneously, making phase synchronization between receivers and phase calibration for each channel essential. In contrast, the proposed method uses a single UWB transceiver and an RF switch to measure only the first-path power at each antenna for direction estimation. Thus, the proposed system can reduce hardware complexity, implementation cost, power consumption, and system size while providing error bounds over the full azimuth range comparable to those of ideal planar PDoA.

## 3. Proposed System Configuration

### 3.1. Proposed Measurement Concept

**Figure 5:**
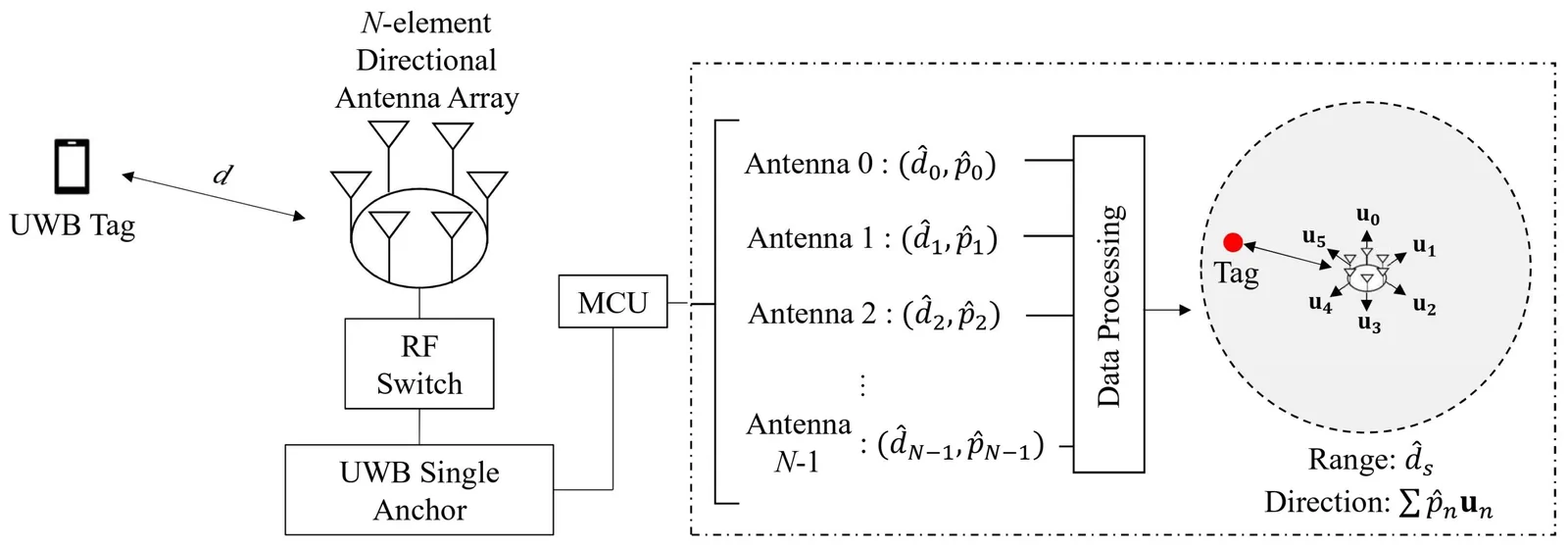
Signal acquisition and processing architecture of the proposed single-anchor UWB localization system.

Figure 5 shows a conceptual diagram of the proposed system. The system consists of a single UWB anchor, an RF switch, and a circular switched antenna array comprising N directional antennas arranged at uniform angular intervals. The RF switch selects one antenna at a time, and the anchor performs one DS-TWR round with the tag during each antenna’s active interval. From the same DS-TWR round, the packet timestamps are used to measure the range, while the first-path power extracted from the CIR of the Final packet is obtained for direction estimation. For an array with N elements, the measurement pair obtained from the n-th antenna after one DS-TWR round is defined as

(10) $$M_{n}=({\hat{d}}_{n},{\hat{p}}_{n}),$$

where ${\hat{d}}_{n}$ is the distance measured through DS-TWR at the n-th antenna, and ${\hat{p}}_{n}$ is the first-path power extracted from the CIR of the packet used in DS-TWR. After all N antennas have been scanned, a measurement set is defined as

(11) $$M=\left\{\left({\hat{d}}_{0},{\hat{p}}_{0}\right),\;\ldots ,\;\left({\hat{d}}_{N-1},{\hat{p}}_{N-1}\right)\right\}.$$

The proposed system estimates distance and direction from this measurement set and combines them to determine the position.

### 3.2. Range Measurement Based on DS-TWR

**Figure 6:**
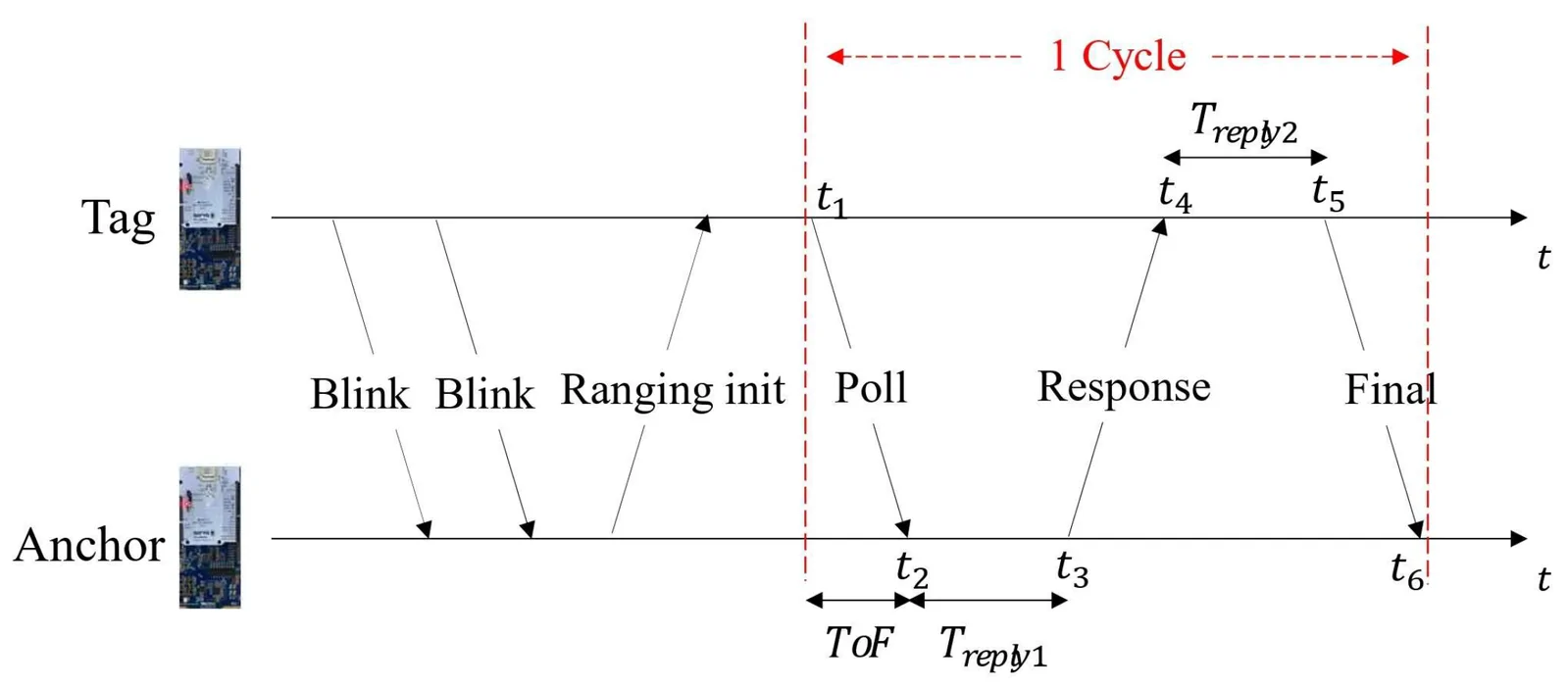
DS-TWR sequence between tag and anchor.

As shown in Figure 6, DS-TWR is performed by sequentially exchanging Poll, Response, and Final packets to estimate the ToF [33]. The tag first completes a brief initialization procedure with the anchor, which is performed only before the first round. It then initiates a DS-TWR round by sending the anchor a Poll packet containing the transmission start timestamp. Upon receiving the Poll packet, the anchor waits for a predefined reply delay before transmitting a Response packet to the tag. Similarly, after receiving the Response packet, the tag waits for a predefined reply delay before transmitting a Final packet to the anchor. The round ends when the anchor receives the Final packet. In Figure 6, $t_{1}$ and $t_{2}$ denote Poll transmission at the tag and reception at the anchor, respectively. Similarly, $t_{3}$ and $t_{4}$ denote Response transmission at the anchor and reception at the tag, while $t_{5}$ and $t_{6}$ denote Final transmission at the tag and reception at the anchor. Each timestamp is measured by the local clock of the corresponding device. The reply delays are $T_{reply1}=t_{3}-t_{2}$ at the anchor and $T_{reply2}=t_{5}-t_{4}$ at the tag.

By combining the round-trip and reply times obtained from the Poll–Response–Final exchange, DS-TWR reduces the effect of the relative clock-frequency offset between the tag and anchor on the ToF estimate. The ToF at the n-th antenna is then calculated as

(12) $${\hat{\tau }}_{n}=\frac{\left(t_{4}-t_{1}\right)\times \left(t_{6}-t_{3}\right)-\left(t_{3}-t_{2}\right)\times \left(t_{5}-t_{4}\right)}{\left(t_{5}-t_{1}+t_{6}-t_{2}\right)}.$$

The corresponding distance estimate is given by

(13) $${\hat{d}}_{n}=c{\hat{\tau }}_{n}-∆d_{n},$$

where c is the speed of light and $∆d_{n}$ is the antenna-specific ranging offset introduced by the RF switch, cable, connector, and antenna path. To reduce random ranging errors associated with individual antenna channels, the final distance estimate ${\hat{d}}_{s}$ for each measurement cycle is calculated by averaging the ranging measurements across all antenna channels as

(14) $${\hat{d}}_{s}=\frac{1}{N}\sum_{n=0}^{N-1}{\hat{d}}_{n}.$$

### 3.3. First-Path Power Extraction

**Figure 7:**
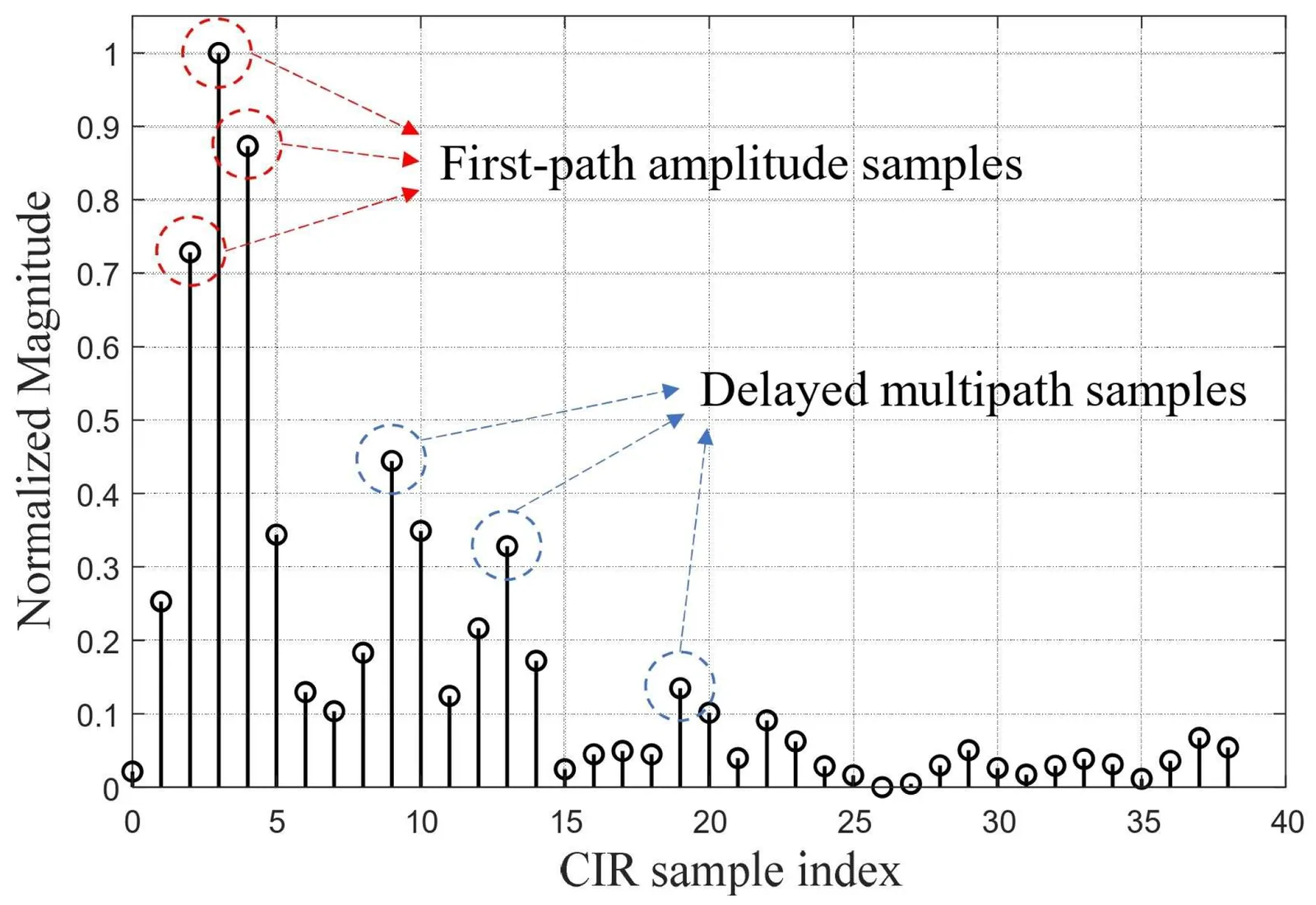
Example of normalized CIR illustrating the first-path amplitude samples used for power estimation and representative delayed multipath samples.

The proposed system estimates the tag’s direction using the first-path power obtained from each of the N antennas to weight the corresponding antenna pointing direction. At each antenna, the power of the tag’s signal used for direction estimation is obtained from the CIR of the Final packet in the DS-TWR exchange. As illustrated in Figure 7, the total CIR power includes contributions from the early response and later components, such as indoor reflections or signals coupled through the switch connections.

These additional contributions can change the relative powers across the antenna channels and affect direction estimation. The proposed system therefore uses three amplitude samples associated with the detected first path to estimate the local early path power while reducing the contribution of temporally separated multipath components. The three-sample calculation follows the DW3000 first-path power estimator, and the first-path power $q_{n}$ in dBm is calculated as

(15) $$q_{n}=10\times log_{10}\left(\frac{F_{1}^{2}+F_{2}^{2}+F_{3}^{2}}{G^{2}}\right)-A,$$

where $F_{1},F_{2},$ and $F_{3}$ are the CIR amplitudes at the first, second, and third sample indices immediately after the first-path position estimated for each received packet, respectively. G is the number of accumulated preamble symbols, which determines the processing gain, and the constant A depends on the pulse repetition frequency (PRF) setting of the physical layer [34].

After compensating for channel-dependent gain differences among the antennas, cables, and switch paths and accounting for noise, the power measured at the n-th antenna is modeled as

(16) $${\hat{p}}_{n}=q_{n}-∆p_{n}+z_{n},$$

where $∆p_{n}$ denotes the RF-path-specific gain correction term in dB, and $z_{n}$ denotes the noise term.

### 3.4. Power-Based Direction Estimation

As described in Section 3.1, the measurement set M, which consists of pairs of range and first-path power measurements obtained from all N antennas, is used for the final position estimation.

Let $\varphi_{n}$ denote the pointing direction of the n-th antenna. The corresponding unit direction vector is defined as

(17) $$u_{n}={\left[\cos\varphi_{n},\sin\varphi_{n}\right]}^{T}.$$

The power measured at each antenna can be used as a weight for the corresponding unit direction vector in (17). Since the sum of the unit direction vectors of the N antennas is zero for a uniform circular array, the power-weighted direction vector is then defined as

(18) $$v=\sum_{n=0}^{N-1}{\hat{p}}_{n}u_{n}.$$

According to (18), the final estimated tag direction is estimated from the resulting vector as

(19) $$\hat{\theta }=atan2(v_{y},v_{x}).$$

where $v_{x}$ and $v_{y}$ are the Cartesian components of v, respectively.

Combining the direction estimate in (19) with the range estimate in (14), the final two-dimensional position is obtained as

(20) $$x={\left[{\hat{d}}_{s}\cos\hat{\theta },{\hat{d}}_{s}\sin\hat{\theta }\right]}^{T}.$$

## 4. System Implementation

### 4.1. Hardware Architecture

**Figure 8:**
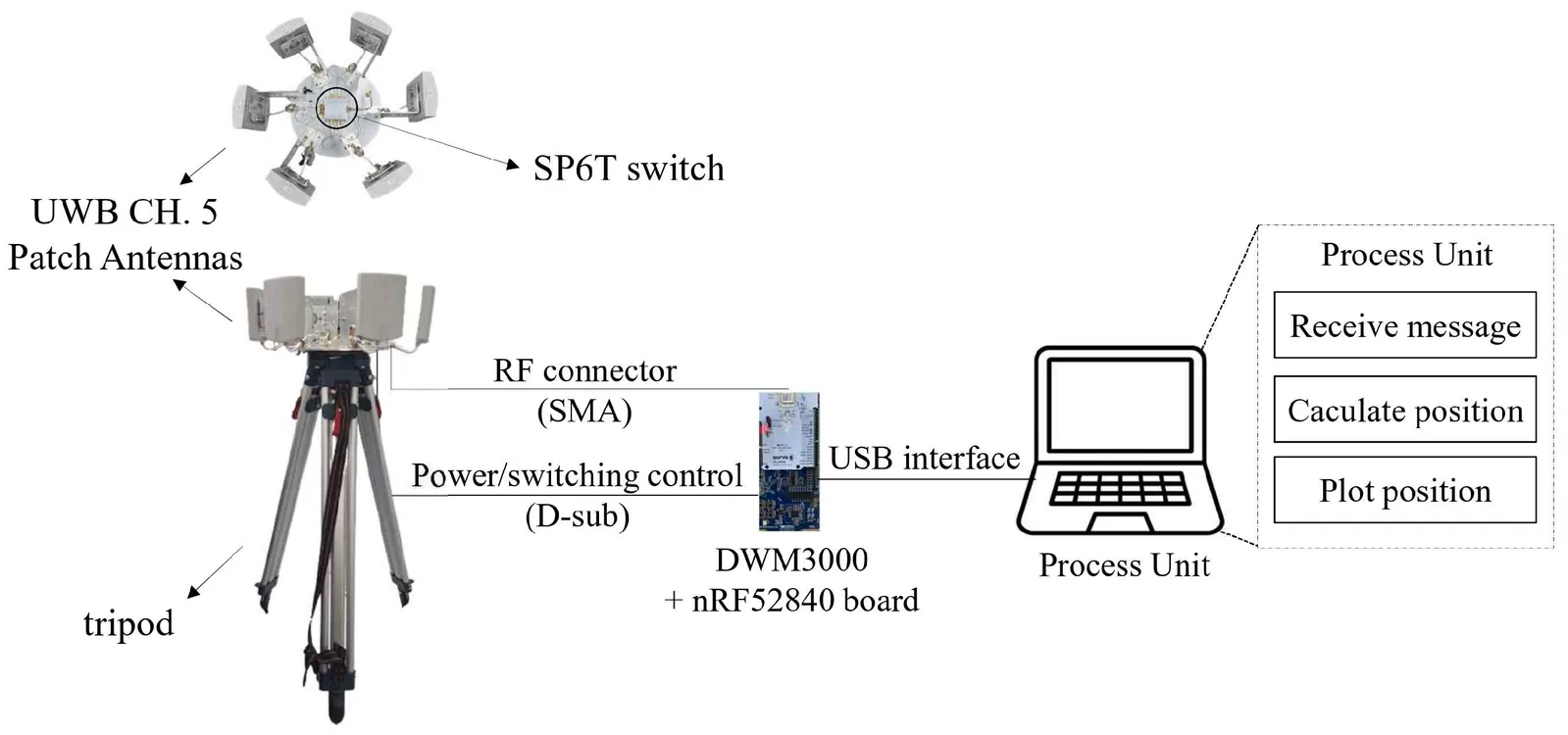
Hardware configuration of the proposed system.

**Figure 9:**
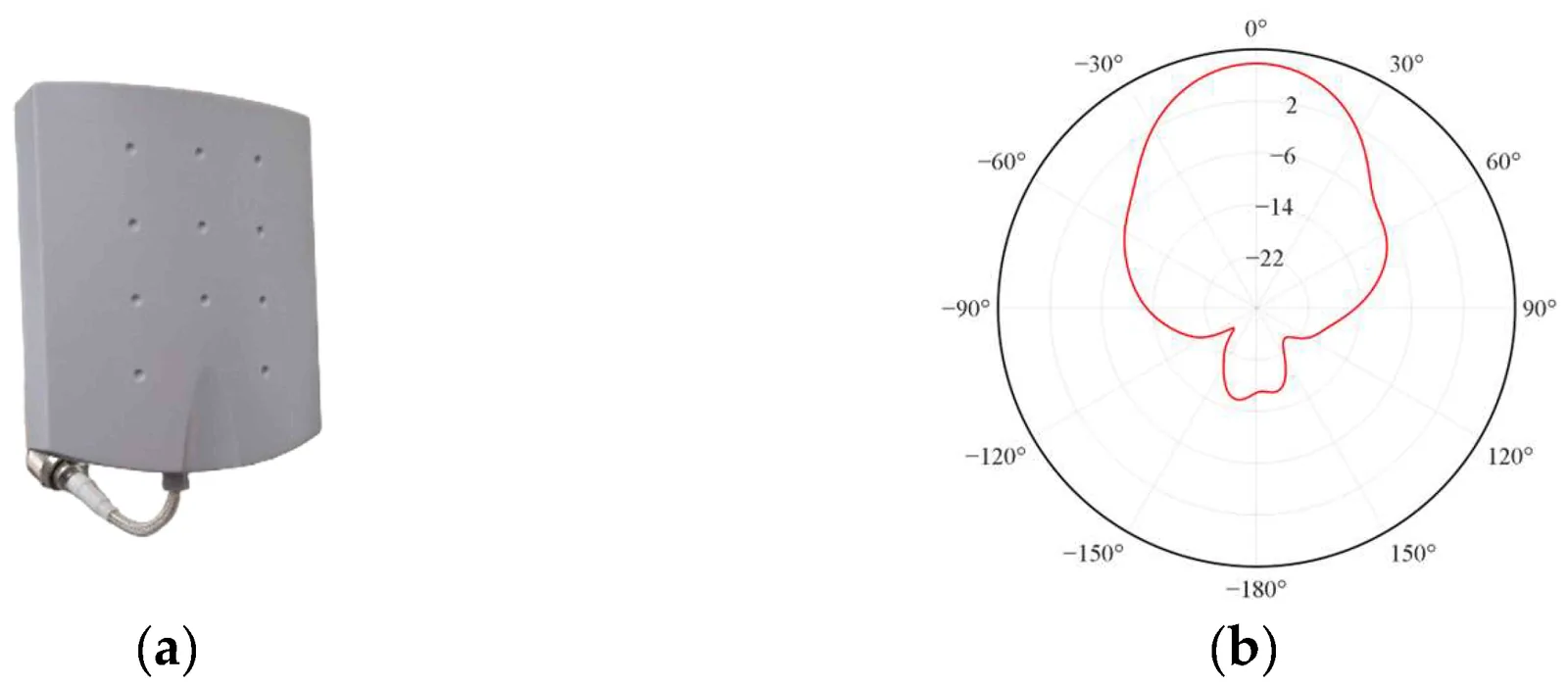
Directional UWB antenna used in the proposed anchor: (**a**) antenna photograph and (**b**) simulated azimuth radiation pattern at the center frequency of UWB channel 5.

To validate the proposed single-anchor UWB RTLS system, the hardware prototype was configured, as shown in Figure 8. The system was implemented using a Qorvo DWM3000 UWB module, an nRF52840 board, six directional antennas, and an SP6T RF switch [35]. Figure 9 shows the directional UWB antenna used in the proposed system and its azimuth radiation pattern at 6.4896 GHz, the center frequency of UWB channel 5. The six directional antennas are arranged in a circular configuration at 60° intervals to provide appropriate coverage of the localization area. The antenna array is mounted on a tripod for stable placement at the center of the localization area. Its elevation angle can also be adjusted to accommodate the antenna radiation pattern and the characteristics of the localization environment.

**Figure 10:**
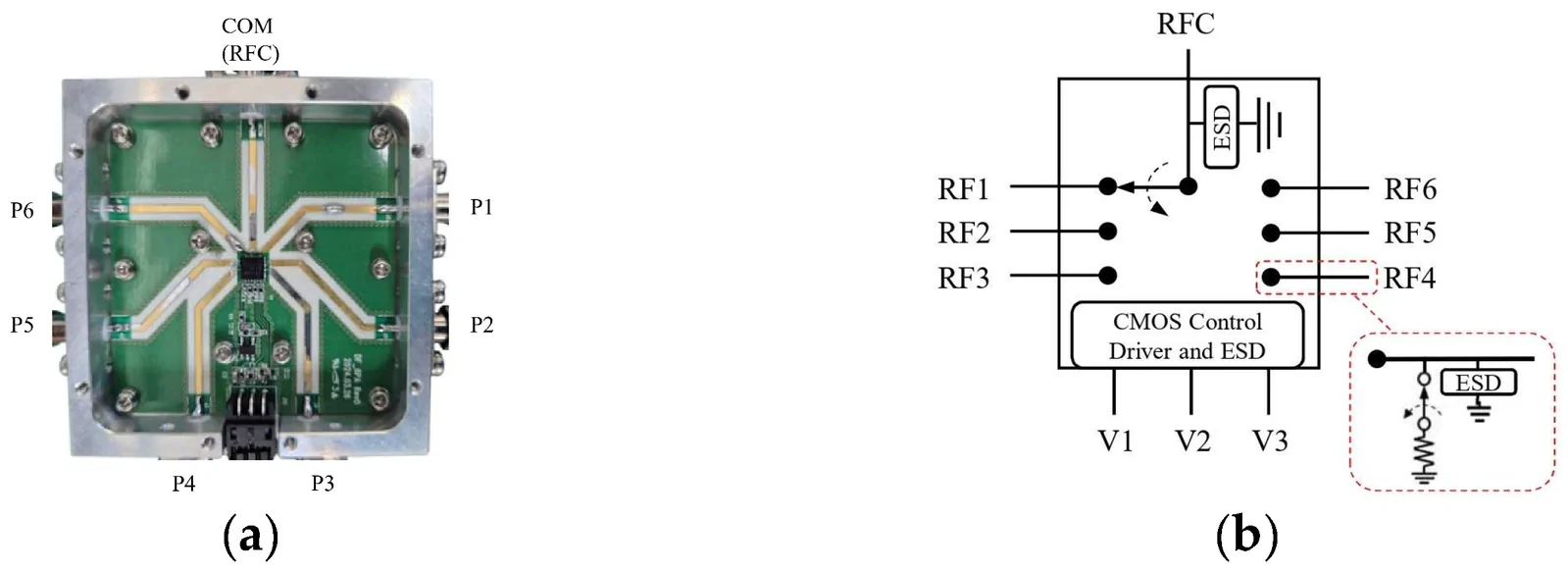
SP6T RF switching circuit used for sequential antenna selection: (**a**) implemented RF switch board and (**b**) simplified circuit configuration based on the PE426462 RF switch IC.

For sequential antenna switching, an UltraCMOS PE426462 SP6T RF switch IC from Peregrine Semiconductor was used, as shown in Figure 10. At the UWB channel-5 center frequency, the measured insertion loss across the six selected RF paths ranged from 1.75 to 1.89 dB, while the isolation between the common port and the unselected ports ranged from 13.21 to 42.39 dB. The common RF port is connected to the DWM3000, while the six RF output ports are connected to the corresponding directional antennas. Whenever a DS-TWR round is completed, or a reception timeout occurs, the control board applies a control signal to the RF switch to sequentially select the active antenna. Accordingly, DS-TWR and power measurements for all six antenna channels can be performed using a single UWB transceiver. After performing DS-TWR with the target tag, the DWM3000 transmits the range and power measurement results for each channel to the host PC. The host PC then estimates the range and direction using the measurement set M and finally calculates the position of the tag.

### 4.2. Measurement and Switching Schedule

**Figure 11:**
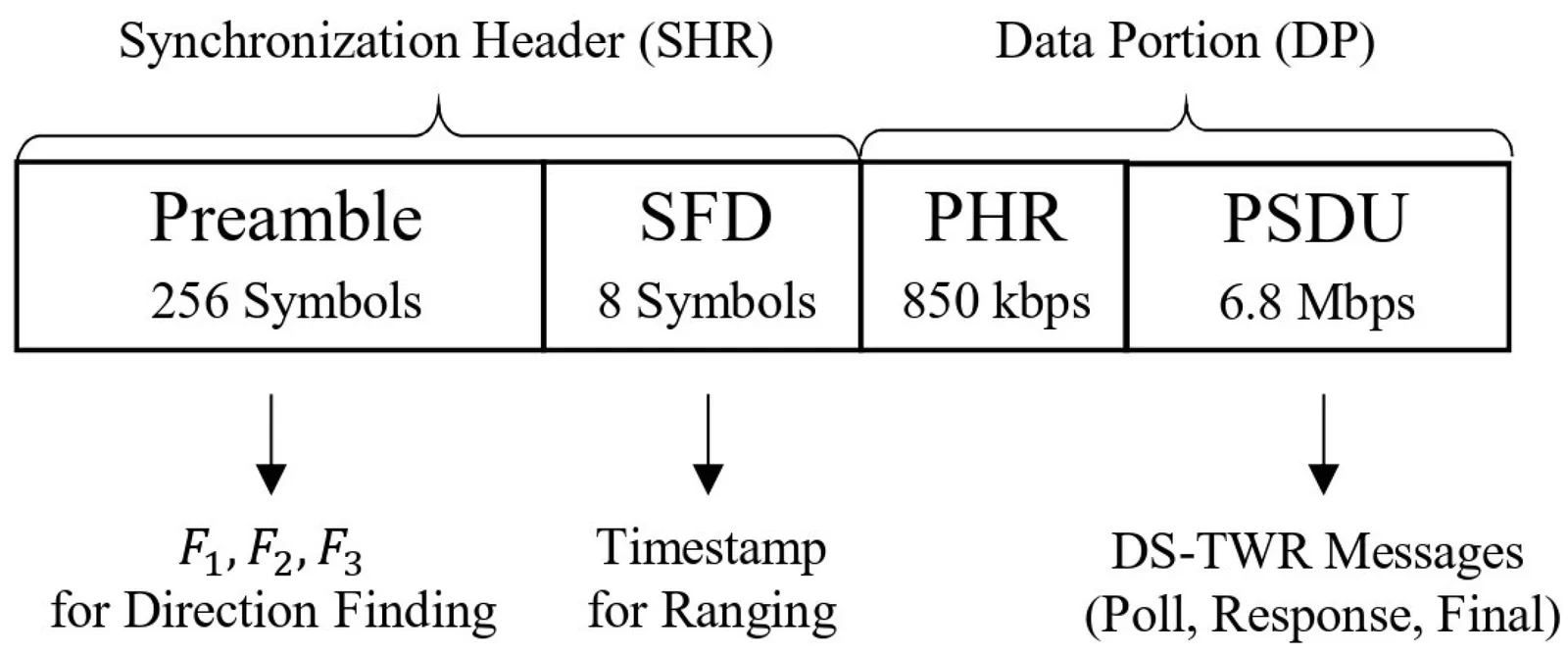
UWB packet structure for DS-TWR ranging and first-path power measurement.

In the proposed system, the single anchor and tag perform one round of DS-TWR for each antenna switching interval. To satisfy both ranging performance and RTLS requirements, an appropriate switching period should be designed by considering the PHY packet length and structure, as well as the processing time required by the embedded system. Figure 11 shows the UWB PHY packet structure used in the proposed system and the function of each field. The synchronization header (SHR) consists of a 256-symbol preamble and an eight-symbol start-of-frame delimiter (SFD), followed by the PHY header (PHR) at 850 kbps and the PHY service data unit (PSDU) at 6.8 Mbps. The $F_{1},\;F_{2},\;F_{3},$ and G values obtained from the received preamble are used for first-path-based power calculation, while the SFD-based timestamp and the payload contained in the PSDU are used for range estimation based on DS-TWR. First-path detection used the manufacturer’s default settings. To distinguish signal arrivals from noise, the receiver derives search thresholds from the measured CIR noise level and estimated noise peaks. The default noise criteria impose threshold lower bounds of 13 times the measured noise level and 1.50 times the estimated noise-peak magnitude. Clock-frequency offset during preamble accumulation can produce low-amplitude replicas, or repeated copies, of the CIR. The receiver uses its clock-offset estimate and preamble code to determine an additional threshold for avoiding these replicas [34].

**Figure 12:**
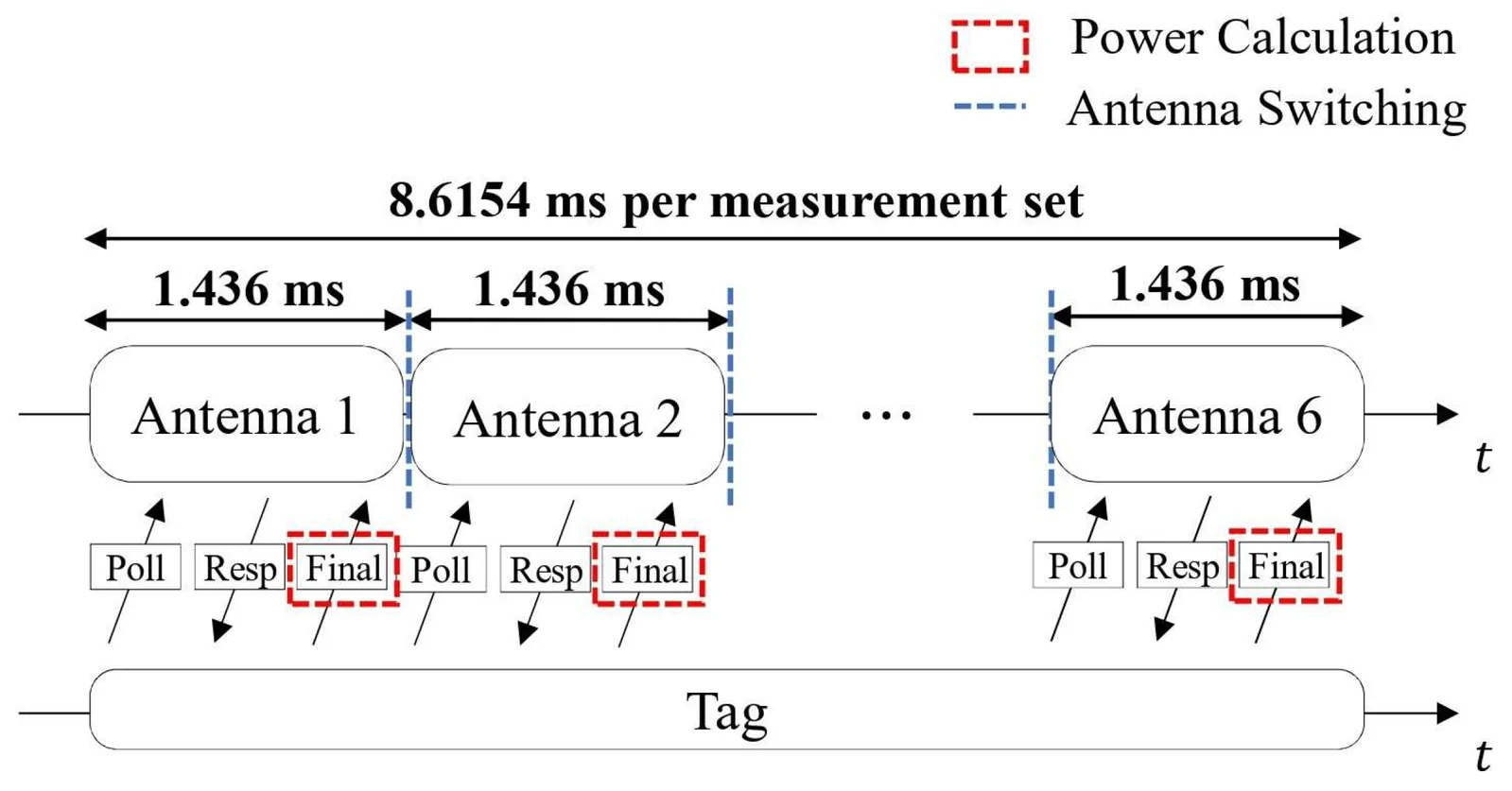
Timing diagram of switched array antenna scheduling scheme.

Figure 12 illustrates the antenna switching period and the overall scheduling scheme designed by considering the processing time and packet length. The duration of one DS-TWR round, $T_{round}$, can be expressed as the sum of the transmission and reception waiting times of the packets and the ToF of each packet. Since the ToF is negligibly small compared with $T_{round}$, the duration of one DS-TWR round is approximated as

(21) $$T_{round}\approx T_{reply1}+T_{reply2}.$$

Considering the processing time and packet length, $T_{reply1}$ and $T_{reply2}$ were set to 923.1 μs and 512.8 μs, respectively. Accordingly, one DS-TWR round was configured to take a total of 1.436 ms, and the reception timeout was set to the same duration. Therefore, neglecting inter-round processing and scheduling overhead, the minimum time required to obtain one measurement set M is approximately 8.6154 ms. This corresponds to a theoretical maximum measurement-set acquisition rate of approximately 116.1 Hz. The measured effective position update rate of the prototype was 103.1 Hz under the experimental conditions. When the active antenna points away from the tag, signal reception may become difficult. To address this issue, the tag retransmits the Poll packet if no Response packet is received during the Response waiting period. Likewise, the anchor switches to the next antenna if no Poll packet is received within the duration of one DS-TWR round.

## 5. Experimental Results and Discussion

**Figure 13:**
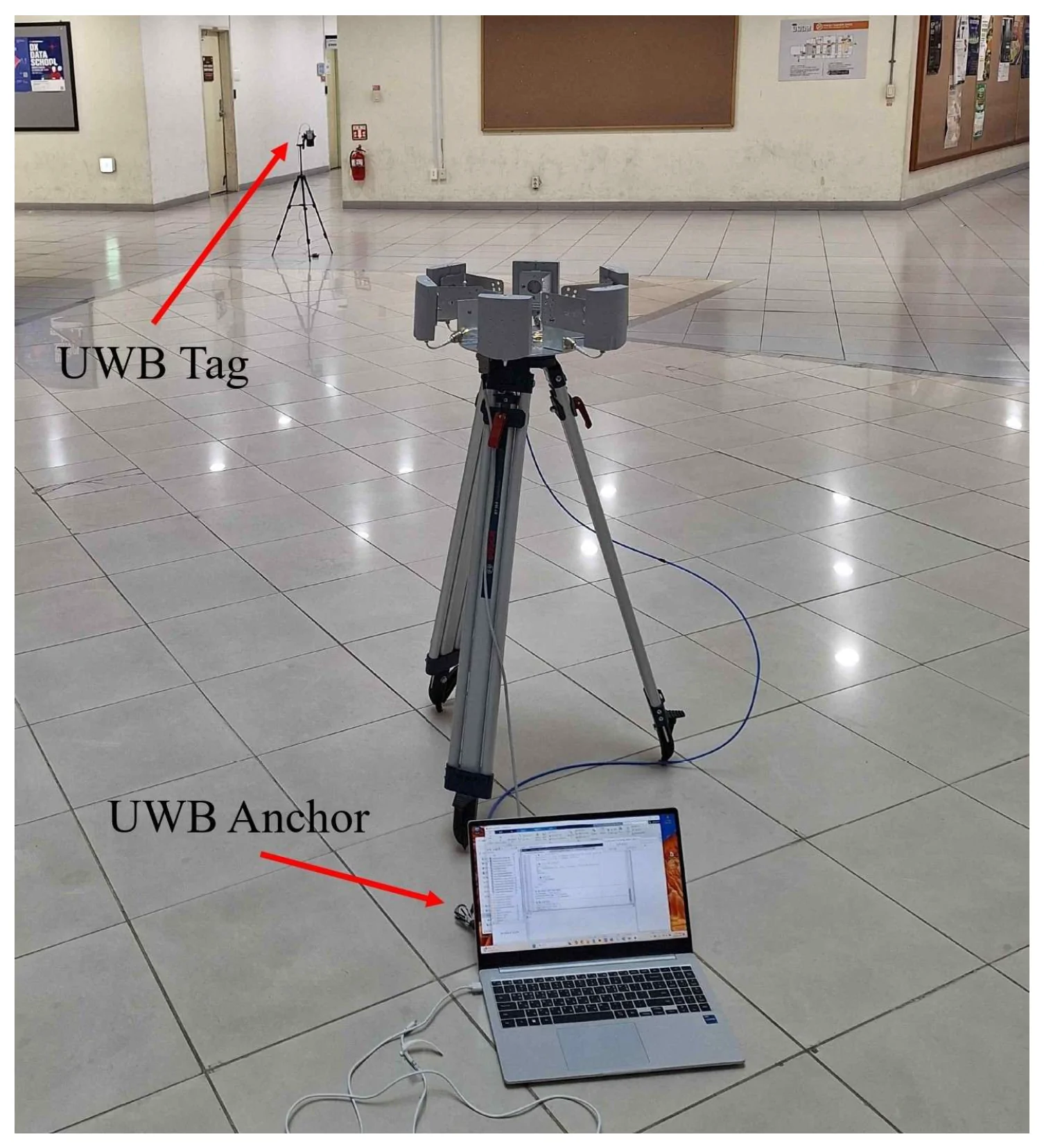
Experimental setup of the proposed switched-beam UWB localization system in an indoor hall.

To validate the proposed system, experiments were conducted as shown in Figure 13. To demonstrate localization capability in a large indoor environment, the experiments were performed in an indoor hall at Kookmin University measuring (12.8 m×22.8 m). Two DWM3000 modules were operated on UWB channel 5 with a center frequency of 6.4896 GHz and a bandwidth of 499.2 MHz.

Prior to the experiments, calibration was performed to compensate for differences among the antenna and RF paths. The tag was placed 5 m from each antenna along its pointing direction, and DS-TWR was repeatedly performed between the anchor and tag for each antenna channel. The measured ranges were compared with the reference distance of 5 m, and the receive RF channel delay of the DWM3000 was adjusted so that the measured distance of each channel matched 5 m, thereby compensating for ranging offsets caused by differences among the switched RF hardware paths. In addition, the first-path power measured under the same conditions was compared to compensate for receive-gain variations among the switched RF hardware paths. This calibration compensates for relative receive-path gain differences, including channel-dependent losses introduced by the RF switch, cables, and connectors. Through this procedure, the RF-path-specific range and power correction values used in the localization experiments were determined.

After calibration, the tag was held stationary at each of 14 reference positions, and 50 position estimates were obtained at each position. To reduce short-term fluctuations in the measured power, a 35-sample centered moving average was applied independently to the power sequence of each antenna channel during post-processing. To examine the contribution of this filtering step, the same dataset was also processed without temporal smoothing. The median and 90th-percentile positioning errors were 0.30 m and 0.71 m without smoothing, compared with 0.24 m and 0.36 m with the 35-sample moving average.

**Figure 14:**
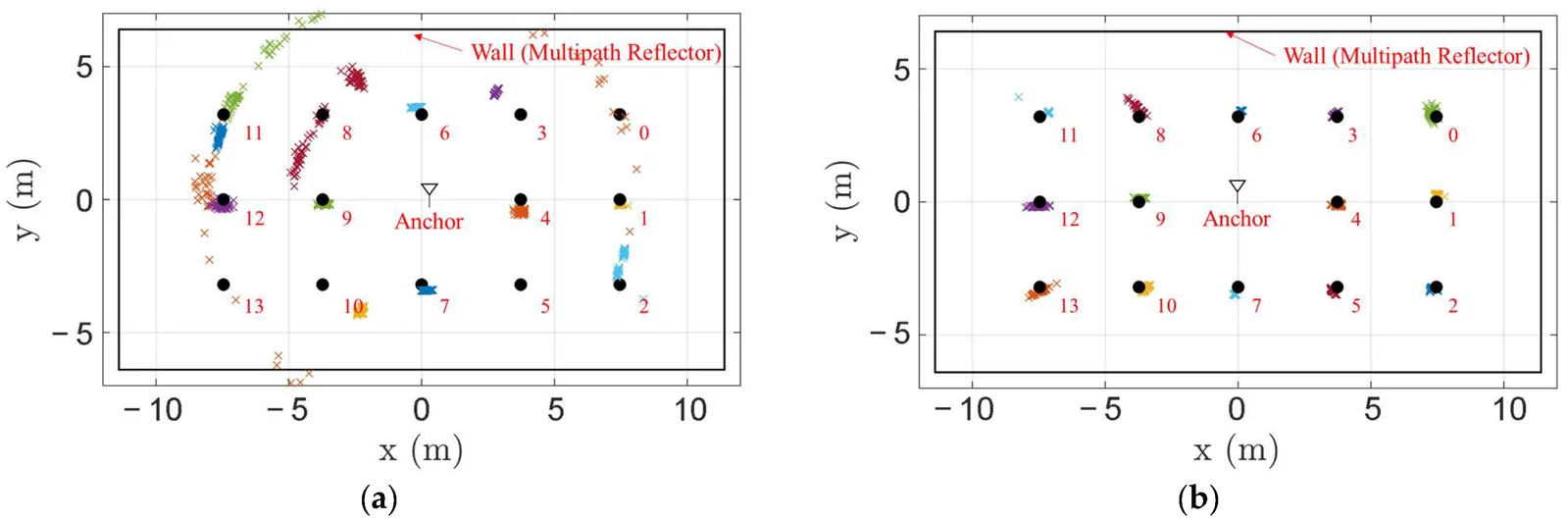
Experimental localization results obtained using (**a**) the total CIR power and (**b**) the first-path power. Black circles indicate the ground-truth positions, and colored markers indicate the estimated tag positions. The numbers indicate the reference position indices.

To evaluate the effect of the power measurement method used for direction estimation, two localization methods were applied to the same CIR measurement data: one using the total CIR power and the other using the first-path power. In both cases, the antenna-specific gain correction, moving-average filtering, range estimation, and power-weighted direction estimation procedures were kept unchanged; only the power values used for direction estimation were varied. The results are shown in Figure 14. As shown in Figure 14a, the localization results obtained using the total CIR power exhibit large errors and substantial scatter at most measurement positions, except for a few locations. This is because the total CIR power includes not only the direct-path component but also delayed multipath components whose power varies across the antenna channels. In contrast, as shown in Figure 14b, the position estimates obtained using the first-path power are concentrated around the corresponding reference positions at most measurement locations.

The measured first-path power can vary when timing errors change the portion of the waveform represented by the three samples. This can occur through an error in the estimated first-path position or a change in the arrival time relative to the sampling instants. If a closely spaced multipath component overlaps those samples, its superposition with the first path also changes the measured power; differences in these effects among antenna channels can then alter the relative powers used for direction estimation.

**Figure 15:**
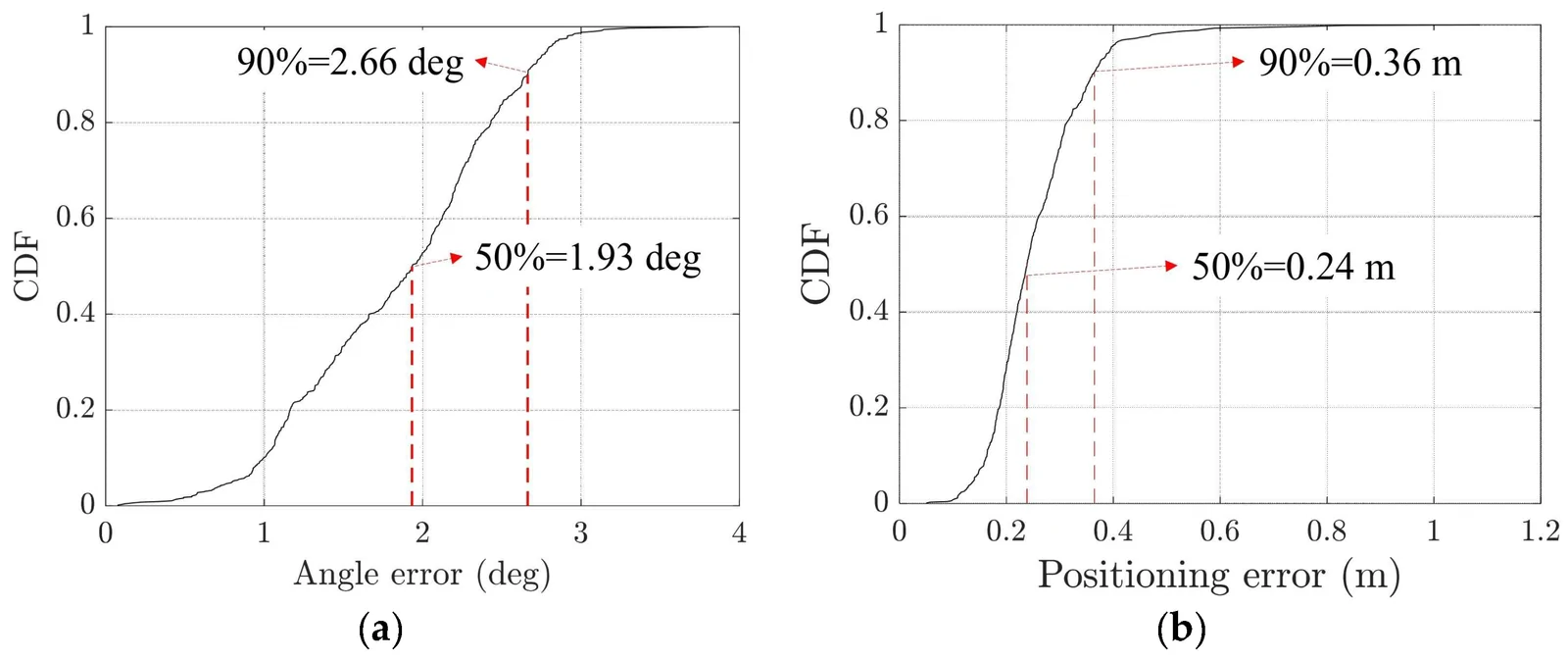
CDFs of the estimation errors using first-path power: (**a**) angle estimation error and (**b**) positioning error.

Figure 15 shows the CDFs of the direction estimation error and the final positioning error obtained using the first-path power. In Figure 15a, the median and 90th-percentile direction estimation errors are 1.93° and 2.66°, respectively. In Figure 15b, the median positioning error is 0.24 m, and the 90th-percentile error is 0.36 m. The error statistics were obtained from 50 repeated estimates at each of 14 reference positions. These repetitions characterize variability at each position, whereas the number and distribution of the reference positions can limit the assessment of distance- and azimuth-dependent performance. The pooled percentiles therefore reflect the particular spatial distribution of the tested positions. These results demonstrate the effectiveness of first-path power for range- and direction-based localization under the tested LoS indoor conditions with multipath propagation. The use of first-path power reduces the contribution of delayed multipath components when the direct path is detectable. Under NLoS conditions, a weakened direct path may fall below the detection threshold, causing a reflected or diffracted component to be detected as the first path. This can bias the direction estimate toward the detected path and increase the measured range; stronger attenuation may also prevent successful ranging. The reported results describe accuracy under the tested LoS indoor conditions.

The six-channel array estimates a continuous direction from the relative first-path powers, with angular sensitivity determined by the beamwidth and overlap of the antenna patterns. Changing the number of antennas changes their angular spacing and the power measurements available for direction estimation; the antenna count therefore needs to be considered together with the radiation patterns and the time required for a complete scan. Differences in the actual antenna radiation patterns can change the relative first-path powers for a given tag direction and introduce angular bias, even after scalar receive-gain calibration. Such calibration compensates for channel gain offsets at the reference condition but does not remove direction-dependent pattern differences. Residual gain mismatch among the switched RF paths can also alter the relative power weights and shift the resulting direction vector. The RF switch introduces insertion loss before the UWB receiver, reducing the received signal level and the margin for detecting weak first paths. Receive-path calibration compensates for relative gain differences, but it does not restore the signal power lost in the switch. In the proposed system, the final range estimate is obtained by taking the arithmetic mean of the range measurements from all antenna channels. This equal weighting retains the symmetry of the circular array in the range estimate. However, the directional antennas can have different link SNRs at the same tag position, and equal weighting gives noisier ranging measurements the same contribution as more reliable measurements. SNR-weighted averaging is a possible extension to reduce the contribution of lower-confidence links. In applying such weighting, the antenna geometry and the existing channel calibration should be considered together to maintain a common range reference at the array center.

The reported results characterize localization accuracy at fixed reference positions. If velocity estimation is required for a moving tag, its azimuthal angular velocity can be estimated from changes in the measured direction over a time interval ∆t. If the two unfiltered direction estimates have independent angular errors with standard deviation $\sigma_{\theta }$, the resulting angular-velocity noise has a standard deviation of $\sigma_{\omega }\approx \sqrt{2}\sigma_{\theta }/∆t$. Temporal smoothing correlates successive estimates and changes this noise relation. The angular-noise contribution to tangential-velocity uncertainty scales with $r\sigma_{\omega }$ and therefore increases with distance. Lower received SNR can further increase angular uncertainty, while changes in multipath or in the detected path under NLoS can produce apparent angular motion unrelated to tag motion.

At a measurement-set rate of 116.1 Hz under successful packet exchanges, consecutive complete scans are separated by approximately 8.6154 ms. Short intervals provide finer temporal resolution but amplify direction noise when velocity is obtained by differencing. The first-path powers are also acquired sequentially within each scan, so motion during acquisition changes the measurement geometry across channels. The 35-sample centered moving average reduces short-term power fluctuations over approximately 0.34 s of data, but can also smooth rapid changes associated with tag motion. The velocity-estimation interval and filtering window therefore need to be chosen together to balance noise suppression and temporal response.

## 6. Conclusions

In this paper, we proposed a single-anchor UWB RTLS system using an array of six directional antennas, a single UWB transceiver, and an RF switch. The proposed system obtains both range and direction information within the same DS-TWR measurement process: the tag range is estimated from the DS-TWR timestamps, while the tag direction is estimated from the relative first-path powers measured across the antenna channels. Using received power instead of inter-channel phase differences for direction estimation, the proposed system simplifies the phase synchronization and calibration required among antenna channels. In addition, because all antennas share a single UWB transceiver, multiple receivers are not required, reducing the power consumption and system size. The use of a single anchor for two-dimensional tag localization also simplifies the system configuration and installation compared with conventional multi-anchor RTLS systems. The proposed architecture mitigates the direction-dependent performance degradation of single-baseline linear-array PDoA while maintaining a constant position error bound over the full azimuth range. In an indoor hall at Kookmin University, 50 samples were collected at each of 14 reference positions. The proposed system achieved median and 90th-percentile direction estimation errors of 1.93° and 2.66°, respectively, and median and 90th-percentile positioning errors of 0.24 m and 0.36 m, respectively, demonstrating sub-meter localization accuracy under the tested stationary-tag LoS indoor conditions. Future work will investigate NLoS operation and multi-tag scheduling. The current prototype supports a single tag; a possible multi-tag extension is to retain the switched-antenna hardware and allocate measurement intervals to individual tags, with a corresponding trade-off in the update rate per tag. Tracking performance will also be evaluated under tag motion, considering the effects of sequential antenna acquisition and temporal filtering.

## Figures and Tables

**Table 1:** Parameters used for the CRLB-based comparison.

Parameters	Value
Center frequency	6.4896 GHz
Bandwidth	499.2 MHz
Signal PSD	−41.3 dBm/MHz
Noise PSD	−113.97 dBm/MHz
Number of observations	64

## Data Availability

The experimental code and MATLAB R2024a script for data processing and analysis used to evaluate the proposed single-anchor UWB localization system are publicly available in the GitHub repository at https://github.com/baeyoonsoo/Single-Anchor-UWB (accessed on 2 November 2026).

## Nomenclature

N	Number of antenna elements
i, n	Antenna indices used in the theoretical analysis and the proposed system, respectively
$N_{eff}$	Effective number of independent observations
r	Distance between the anchor and tag
θ	Tag azimuth; incidence angle measured from broadside for the linear-array analysis
k, λ	Wavenumber and signal wavelength, respectively
$d_{a}$	Antenna element spacing
ρ(r), $\rho_{i}(\theta ,r)$	Received SNR at distance r and at the i-th antenna, respectively
A(r)	Received-power factor accounting for distance-dependent path loss and receiver gain
$G_{i}(\theta )$	Gain of the i-th antenna at the tag azimuth
$G_{max}$	Maximum antenna gain
$\Theta_{HPBW}$	Half-power beamwidth
$\varphi_{i}$, $\varphi_{n}$	Antenna pointing directions in the theoretical analysis and the proposed system, respectively
${∆}_{i}\left(\theta \right)$	Wrapped angular difference between the tag direction and the i-th antenna pointing direction
$\;{\hat{P}}_{i}\left(\theta ,r\right)$	Received-power observation at the i-th antenna in the theoretical model
$P_{i}\left(\theta ,r\right)$	Received-power model value used in the CRLB analysis
$n_{i}$, $\sigma_{P,i}^{2}$	Power measurement error in the theoretical model and its variance, respectively
${CRLB}_{PDoA}\left(\theta \right)$	Angular variance bounds for PDoA-based estimation
${CRLB}_{Power}\left(\theta \right)$	Angular variance bounds for power-based estimation
${CRLB}_{\theta }$	Angular variance bound used to calculate the position error bound
$\sigma_{∆\varphi }$	Standard deviation of the residual phase-difference error
${MSE}_{\theta ,PDoA}\left(r,\theta \right)$	Local angular mean-square error prediction for nonideal PDoA
PEB(r,θ)	Two-dimensional position error bound
$\sigma_{r}$	Standard deviation of the TWR ranging error
$M_{n}$, M	Range-power measurement pair for the *n*-th antenna and the complete measurement set, respectively
${\hat{d}}_{n}$	DS-TWR distance estimate for the *n*-th antenna
${\hat{p}}_{n}$	Gain-corrected first-path power used for direction estimation
$t_{1}$ *,* $t_{2}$	Poll transmission timestamp at the tag and reception timestamp at the anchor, respectively
$t_{3}$ *,* $t_{4}$	Response transmission timestamp at the anchor and reception timestamp at the tag, respectively
$t_{5}$ *,* $t_{6}$	Final transmission timestamp at the tag and reception timestamp at the anchor, respectively
$T_{reply1}$, $T_{reply2}$	Reply delays at the anchor and tag, respectively
${\hat{\tau }}_{n}$	Estimated time of flight for the *n*-th antenna
c	Speed of light
$∆d_{n}$	Antenna-specific ranging offset
${\hat{d}}_{s}$	Final range estimate obtained by averaging the antenna-channel range estimates
$q_{n}$	First-path power calculated from the CIR amplitude samples (dBm)
$F_{1},F_{2}$, $F_{3}$	Three CIR amplitude samples immediately after the detected first-path position
G	Number of accumulated preamble symbols
A	PRF-dependent constant in the DW3000 first-path power calculation
$∆p_{n}$	RF-path-specific power correction term (dB)
$z_{n}$	Noise term in the corrected first-path power model
$u_{n}$	Unit vector in the pointing direction of the n-th antenna
v	Power-weighted direction vector
$v_{x}$, $v_{y}$	Cartesian components of the power-weighted direction vector
$\hat{\theta }$	Estimated tag azimuth
x	Estimated two-dimensional tag position
$T_{round}$	Duration of one DS-TWR round
∆t	Time interval between two direction estimates
$\sigma_{\theta }$	Standard deviation of the unfiltered angular estimation error
$\sigma_{\omega }$	Standard deviation of the angular-velocity noise
